# Supplementary material for: Identification of Endometrial Cancer-Specific microRNA Biomarkers in Endometrial Fluid
Source: Int J Mol Sci. 2023 May 12;24(10):8683. doi: 10.3390/ijms24108683 (PMC10218319; doi:10.3390/ijms24108683)
Supplement: Supplementary file 1 [file ijms-24-08683-s001.zip › Supplementary S1_Method.pdf]

Supplemental Materials

Supplementary S1_Method

Endometrial fluid sample collection occurred upon patient consent, either in the clinic or operating room. A clean speculum was inserted into the vagina and used to visualize the cervix. When performed in the clinic, the cervix was sanitized using a swab saturated with betadine solution using a single pass circumferentially across the surface of the cervix. The collection was done prior to prep without sanitation when performed in the operating room. A sterile SIS catheter (Goldstein SonoBiopsy Catheter, Cook Medical LLC, Bloomington, IN, USA) was set to a default length of 5.5 cm for acorn placement. This was passed through the external cervical os and into the uterine cavity. If unable to be passed by itself, a single tooth tenaculum was used to assist with passage by applying the tenaculum to the cervix for traction. When needed, cervical dilators were employed to sequentially dilate the cervix to 8 French (2.5 mm) to allow passage of the catheter. Six mL of sterile saline was slowly injected into the endometrial cavity and aspirated using a 10 mL sterile syringe. The injection was repeated using the same fluid to inject and aspirate up to three times, collecting as much fluid as possible with the final aspiration. If difficulties were encountered with fluid aspiration, any spontaneously expelled fluid present in the vaginal vault was collected. The collected fluid was placed into a sterile Nunc 15mL Conical Polypropylene Centrifuge Tube and placed on wet ice immediately, with further processing to take place within one hour of collection. Upon completion, the catheter and all other instruments were removed. The planned surgical procedure was performed either immediately or as scheduled, consistent with the standard of care, with routine specimen processing and histologic diagnosis.
