# Supplementary material for: Identification of Endometrial Cancer-Specific microRNA Biomarkers in Endometrial Fluid
Source: Int J Mol Sci. 2023 May 12;24(10):8683. doi: 10.3390/ijms24108683 (PMC10218319; doi:10.3390/ijms24108683)
Supplement: Supplementary file 1 [file ijms-24-08683-s001.zip › Supplementary S2_Data Analysis.pdf]

Supplementary S2 – Data Analysis

Data obtained from miScript miRNA PCR Array was uploaded and analyzed using QIAGEN Data Analysis Protocol on their web resources: The GeneGlobe Data Analysis Center. The miScript miRNA PCR Data Analysis spreadsheet can be found under "Product Resources/Performance Data" at www.qiagen.com/us/shop/pcr/primer-sets/miscript-mirna-pcr-arrays/#resources, once the user was registered.
