# Supplementary material for: Identification of Endometrial Cancer-Specific microRNA Biomarkers in Endometrial Fluid
Source: Int J Mol Sci. 2023 May 12;24(10):8683. doi: 10.3390/ijms24108683 (PMC10218319; doi:10.3390/ijms24108683)
Supplement: Supplementary file 1 [file ijms-24-08683-s001.zip › Table S4_Custom CMIHS02413_Actual Final Plate Gene Layout.pdf]

Product Specification Sheet

miScript™ miRNA PCR Array: CMIHS02413
Custom miScript™ miRNA PCR Array

Array Layout

	1	2	3	4	5	6	7	8	9	10	11	12
A	hsa-miR-200c-3p	hsa-miR-210-3p	hsa-miR-203a-3p	hsa-miR-218-5p	hsa-miR-183-5p	hsa-miR-185-3p	hsa-miR-185-5p	hsa-miR-429	hsa-miR-146a-5p	hsa-miR-17-5p	hsa-miR-18a-3p	hsa-miR-21-5p
B	hsa-let-7c-5p	hsa-miR-7-5p	hsa-miR-200a-3p	hsa-miR-200b-3p	hsa-miR-205-5p	hsa-miR-181a-5p	hsa-miR-182-5p	hsa-miR-135a-5p	hsa-miR-449a	hsa-miR-423-5p	hsa-miR-103a-3p	hsa-miR-107
C	hsa-miR-34b-3p	hsa-miR-9-5p	hsa-miR-27a-5p	hsa-miR-17-5p	hsa-miR-126-3p	hsa-miR-126-5p	hsa-miR-199b-5p	hsa-miR-203a-3p	hsa-miR-204-5p	hsa-miR-221-3p	hsa-miR-221-5p	hsa-let-7a-5p
D	hsa-let-7e-5p	hsa-miR-15b-5p	hsa-miR-20a-5p	hsa-miR-30c-5p	hsa-miR-34b-3p	hsa-miR-99b-5p	hsa-miR-106b-5p	hsa-miR-152-3p	hsa-miR-192-5p	hsa-miR-193a-3p	hsa-miR-222-3p	hsa-miR-410-3p
E	hsa-miR-424-5p	hsa-miR-1247-3p	hsa-miR-370-3p	hsa-miR-376c-3p	hsa-miR-542-5p	hsa-miR-377-3p	hsa-miR-362-3p	hsa-miR-223-3p	hsa-miR-10a-5p	hsa-miR-32-5p	hsa-miR-124-3p	hsa-miR-125b-5p
F	hsa-miR-19b-3p	hsa-miR-4705	hsa-miR-34a-5p	hsa-miR-155-5p	hsa-miR-16-5p	hsa-miR-124-3p	hsa-miR-296-5p	hsa-miR-199a-5p	hsa-miR-335-5p	hsa-miR-188-5p	hsa-miR-378c	hsa-miR-1228-3p
G	hsa-miR-3200-5p	hsa-miR-3613-3p	hsa-miR-1-3p	hsa-miR-196a-5p	hsa-miR-1207-3p	hsa-miR-1321	hsa-miR-128-3p	hsa-miR-3918	hsa-miR-4685-5p	hsa-miR-149-5p	hsa-miR-7110-3p	hsa-miR-149-3p
H	miRTC	PPC	miRTC	PPC	miRTC	PPC	SNORD61	SNORD68	SNORD61	SNORD68	cel-miR-39-3p	cel-miR-39-3p

Gene Table

Position	MatureSangerID	MatureSangerAccession	Catalog Number
A01	hsa-miR-200c-3p	MIMAT0000617	MS00003752
A02	hsa-miR-210-3p	MIMAT0000267	MS00003801
A03	hsa-miR-203a-3p	MIMAT0000264	MS00003766
A04	hsa-miR-218-5p	MIMAT0000275	MS00006769
A05	hsa-miR-183-5p	MIMAT0000261	MS00031507
A06	hsa-miR-185-3p	MIMAT0004611	MS00008876
A07	hsa-miR-185-5p	MIMAT0000455	MS00003647
A08	hsa-miR-429	MIMAT0001536	MS00004193
A09	hsa-miR-146a-5p	MIMAT0000449	MS00003535
A10	hsa-miR-17-5p	MIMAT0000070	MS00029274
A11	hsa-miR-18a-3p	MIMAT0002891	MS00006538
A12	hsa-miR-21-5p	MIMAT0000076	MS00009079
B01	hsa-let-7c-5p	MIMAT0000064	MS00003129
B02	hsa-miR-7-5p	MIMAT0000252	MS00032116
B03	hsa-miR-200a-3p	MIMAT0000682	MS00003738
B04	hsa-miR-200b-3p	MIMAT0000318	MS00009016
B05	hsa-miR-205-5p	MIMAT0000266	MS00003780
B06	hsa-miR-181a-5p	MIMAT0000256	MS00008827
B07	hsa-miR-182-5p	MIMAT0000259	MS00008855
B08	hsa-miR-135a-5p	MIMAT0000428	MS00008624
B09	hsa-miR-449a	MIMAT0001541	MS00004228
B10	hsa-miR-423-5p	MIMAT0004748	MS00009681
B11	hsa-miR-103a-3p	MIMAT0000101	MS00031241
B12	hsa-miR-107	MIMAT0000104	MS00031255
C01	hsa-miR-34b-3p	MIMAT0004676	MS00008190
C02	hsa-miR-9-5p	MIMAT0000441	MS00010752
C03	hsa-miR-27a-5p	MIMAT0004501	MS00009240
C04	hsa-miR-17-5p	MIMAT0000070	MS00029274
C05	hsa-miR-126-3p	MIMAT0000445	MS00003430
C06	hsa-miR-126-5p	MIMAT0000444	MS00006636
C07	hsa-miR-199b-5p	MIMAT0000263	MS00003731
C08	hsa-miR-203a-3p	MIMAT0000264	MS00003766
C09	hsa-miR-204-5p	MIMAT0000265	MS00003773
C10	hsa-miR-221-3p	MIMAT0000278	MS00003857

Position	MatureSangerID	MatureSangerAccession	Catalog Number
C11	hsa-miR-221-5p	MIMAT0004568	MS00009170
C12	hsa-let-7a-5p	MIMAT0000062	MS00031220
D01	hsa-let-7e-5p	MIMAT0000066	MS00031227
D02	hsa-miR-15b-5p	MIMAT0000417	MS00008792
D03	hsa-miR-20a-5p	MIMAT0000075	MS00003199
D04	hsa-miR-30c-5p	MIMAT0000244	MS00009366
D05	hsa-miR-34b-3p	MIMAT0004676	MS00008190
D06	hsa-miR-99b-5p	MIMAT0000689	MS00032165
D07	hsa-miR-106b-5p	MIMAT0000680	MS00003402
D08	hsa-miR-152-3p	MIMAT0000438	MS00003591
D09	hsa-miR-192-5p	MIMAT0000222	MS00003689
D10	hsa-miR-193a-3p	MIMAT0000459	MS00031542
D11	hsa-miR-222-3p	MIMAT0000279	MS00007609
D12	hsa-miR-410-3p	MIMAT0002171	MS00004144
E01	hsa-miR-424-5p	MIMAT0001341	MS00004186
E02	hsa-miR-1247-3p	MIMAT0022721	MS00042357
E03	hsa-miR-370-3p	MIMAT0000722	MS00004053
E04	hsa-miR-376c-3p	MIMAT0000720	MS00004046
E05	hsa-miR-542-5p	MIMAT0003340	MS00007147
E06	hsa-miR-377-3p	MIMAT0000730	MS00004095
E07	hsa-miR-362-3p	MIMAT0004683	MS00009562
E08	hsa-miR-223-3p	MIMAT0000280	MS00003871
E09	hsa-miR-10a-5p	MIMAT0000253	MS00031262
E10	hsa-miR-32-5p	MIMAT0000090	MS00003297
E11	hsa-miR-124-3p	MIMAT0000422	MS00006622
E12	hsa-miR-125b-5p	MIMAT0000423	MS00006629
F01	hsa-miR-19b-3p	MIMAT0000074	MS00031584
F02	hsa-miR-4705	MIMAT0019805	MS00039928
F03	hsa-miR-34a-5p	MIMAT0000255	MS00003318
F04	hsa-miR-155-5p	MIMAT0000646	MS00031486
F05	hsa-miR-16-5p	MIMAT0000069	MS00031493
F06	hsa-miR-124-3p	MIMAT0000422	MS00006622
F07	hsa-miR-296-5p	MIMAT0000690	MS00016401
F08	hsa-miR-199a-5p	MIMAT0000231	MS00006741
F09	hsa-miR-335-5p	MIMAT0000765	MS00003976
F10	hsa-miR-188-5p	MIMAT0000457	MS00003668
F11	hsa-miR-378c	MIMAT0016847	MS00021119
F12	hsa-miR-1228-3p	MIMAT0005583	MS00042385
G01	hsa-miR-3200-5p	MIMAT0017392	MS00022925
G02	hsa-miR-3613-3p	MIMAT0017991	MS00031787
G03	hsa-miR-1-3p	MIMAT0000416	MS00008358
G04	hsa-miR-196a-5p	MIMAT0000226	MS00031563
G05	hsa-miR-1207-3p	MIMAT0005872	MS00014182
G06	hsa-miR-1321	MIMAT0005952	MS00014644
G07	hsa-miR-128-3p	MIMAT0000424	MS00008582
G08	hsa-miR-3918	MIMAT0018192	MS00023576
G09	hsa-miR-4685-5p	MIMAT0019771	MS00040117
G10	hsa-miR-149-5p	MIMAT0000450	MS00003570
G11	hsa-miR-7110-3p	MIMAT0028118	MS00048265
G12	hsa-miR-149-3p	MIMAT0004609	MS00037702
H01	miRTC	SA_miRNA_005	MS00000001
H02	PPC	SA_00104	MS00000002
H03	miRTC	SA_miRNA_005	MS00000001
H04	PPC	SA_00104	MS00000002
H05	miRTC	SA_miRNA_005	MS00000001
H06	PPC	SA_00104	MS00000002
H07	SNORD61	NR_002735	MS00033705
H08	SNORD68	NR_002450	MS00033712
H09	SNORD61	NR_002735	MS00033705
H10	SNORD68	NR_002450	MS00033712
H11	cel-miR-39-3p	MIMAT0000010	MS00019789
H12	cel-miR-39-3p	MIMAT0000010	MS00019789
